# Preparation and Characterization of *Brassica rapa* L. Polysaccharide–Zein Nanoparticle Delivery System Loaded with Capsaicin

**DOI:** 10.3390/molecules30224459

**Published:** 2025-11-19

**Authors:** Mi Yuan, Lele Chen, Hamulati Hasimu, Mengying Hu, Xiaojun Yang

**Affiliations:** 1 College of Food and Pharmaceutical Science, Xinjiang Agricultural University, Urumqi 836500, China; yuanmi0823@163.com (M.Y.); chenll1217@163.com (L.C.); 2 Xinjiang Institute of Materia Medica, Urumqi 830034, China; Hamulati@sina.com (H.H.); mengying1105@sohu.com (M.H.)

**Keywords:** *Brassica rapa* L. polysaccharide, capsaicin, nanoparticle, characterization, anti-inflammatory, xylene-induced ear edema

## Abstract

Capsaicin, a natural bioactive compound, has attracted wide interest for its potential health benefits. However, its rapid metabolism and strong irritancy upon oral administration have greatly limited its further application. To address these issues, this study developed a nanoparticle delivery system using corn Zein and *Brassica rapa* L. polysaccharide (BP) as carriers, with capsaicin (CAP) as the core. The optimized formulation (BP:Zein = 1:2, Zein:CAP = 2.5:1, mg/mg) produced stable, uniform spherical nanoparticles with an average particle size of 203.05 nm, a polydispersity index (PDI) of 0.138, a zeta potential of −44.9 mV, an encapsulation efficiency of 54.03%, and a drug loading capacity of 184.57 μg/mg. Fourier transform infrared spectroscopy (FTIR), fluorescence spectroscopy (FS), X-Ray diffraction, scanning electron microscope (SEM), and transmission electron microscopy (TEM) analyses confirmed that CAP was successfully encapsulated, forming nanoparticles through hydrogen bonding and hydrophobic interactions between CAP and Zein. The obtained nanoparticles displayed regular spherical morphology and uniform size distribution. Compared with single-layer Zein–CAP nanoparticles, BP–Zein–Capsaicin (BZC) nanoparticles exhibited markedly improved stability under different pH, ionic strength, and storage conditions. In vitro simulated digestion showed a sustained-release profile, with 36.76% of CAP released after 4 h. The anti-inflammatory experiment showed that both the nanoparticle and free capsaicin groups significantly inhibited xylene-induced acute ear edema in mice, with the medium- and high-dose nanoparticle groups exhibiting stronger anti-inflammatory effects than the free capsaicin group. These findings suggest that the nanoparticle delivery system effectively enhances the anti-inflammatory activity of capsaicin, possibly by improving its stability, achieving sustained release, and enhancing its bioavailability in vivo. Overall, capsaicin-loaded *Brassica rapa* L. polysaccharide–Zein nanoparticles combine small particle size, high drug loading, and excellent stability, providing a promising strategy for functional food development and targeted bioactive delivery.

## 1. Introduction

**Figure 1:**
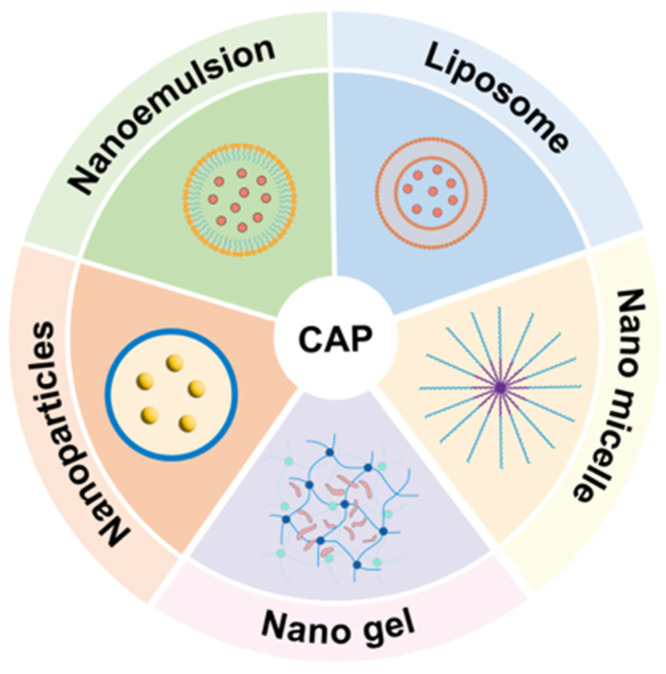
Capsaicin delivery carrier.

Capsaicin (CAP), a natural compound extracted from chili fruits, is the principal pungent component and is classified as an alkaloid [1]. More than 30 capsaicinoid compounds have been identified, among which capsaicin and dihydrocapsaicin account for approximately 90% of the total content [2]. Due to its various bioactivities, including analgesic [3], anti-inflammatory [4], antioxidant [5], and anticancer properties [6], CAP has attracted considerable attention. However, its strong pungency, rapid metabolism, and short half-life after oral administration limit its therapeutic potential. Direct oral intake of CAP causes adverse effects such as oral inflammation, gastric ulcer, and gastrointestinal spasms [7]. Encapsulation strategies can reduce its irritancy, prolong its half-life, and improve absorption by controlling its release in the gastrointestinal tract [8]. Reported delivery systems for CAP include liposomes [9], nanocrystals [10], nanoemulsions [11], and nanotubes [12] (Figure 1). Nevertheless, oral delivery systems with sustained-release properties remain relatively limited.

*Brassica rapa* L. is a biennial herb of the Cruciferae family, widely distributed in high-altitude regions such as Xinjiang and Tibet, China. According to the Chinese Materia Medica, it is pungent, sweet, and bitter in taste, with a warm nature. Phytochemical studies have shown that *Brassica rapa* L. contains saponins, flavonoids, polysaccharides, and alkaloids [13]. Among them, *Brassica rapa* L. polysaccharide (BP) has demonstrated antitumor [14], immunomodulatory [15], anti-inflammatory, and hypoglycemic activities [16], showing great potential in functional food development.

Zein, a prolamin protein derived from corn, is an inexpensive and environmentally friendly by-product of corn processing. With distinct hydrophilic and hydrophobic domains, Zein molecules readily self-assemble and possess unique solubility characteristics, making Zein an ideal material for fabricating nanocarriers for hydrophobic compounds. Zein-based nanoparticles have been extensively investigated for the encapsulation of drugs, nutrients, phytochemicals, enzymes, essential oils, and antioxidants [17]. However, while Zein nanoparticles offer notable advantages as delivery vehicles, their poor stability under adverse physicochemical conditions and susceptibility to enzymatic degradation during digestion significantly restrict their protective function [18]. This limitation highlights the need for composite nanocarriers that integrate Zein with other biopolymers to achieve improved stability and enhanced efficacy.

Nanoparticles (NPs) are commonly employed as carriers for bioactive compounds or natural nutrients in food delivery systems, facilitating targeted nutrient delivery and significantly enhancing their gastrointestinal absorption and bioavailability. Polysaccharides and proteins derived from animals, plants, or microorganisms are considered safe and biocompatible carriers [19]. Their abundant functional groups and modifiability make polysaccharides particularly attractive for constructing nanocarriers [20]. Among the fabrication techniques, the anti-solvent precipitation method is the most widely applied in the food field. By introducing an antisolvent, the system reaches a supersaturated state, inducing molecular self-assembly into nanoparticles [21]. In practice, hydrophobic solutes, such as biopolymer molecules or functional bioactive compounds, are first dissolved in a polar organic solvent, such as ethanol, methanol, or acetone, to form a homogeneous binary solution. This solution is then slowly added to a specified amount of anti-solvent, typically water, which must be fully miscible with the polar solvent. As the concentration of the anti-solvent increases, the system gradually undergoes phase separation, causing the solute to precipitate and self-assemble into nanoparticles. In this study, this method was employed to prepare nanoparticles [22].

The present study aimed to investigate the particle size, polydispersity index (PDI), and zeta potential of nanoparticles prepared with different wall-to-core ratios, to evaluate the encapsulation efficiency, drug loading, and environmental stability of capsaicin-loaded *Brassica rapa* L. polysaccharide–Zein nanoparticles, and to characterize their structural properties using SEM, FTIR, fluorescence spectroscopy, XRD, and TEM. In addition, the in vivo anti-inflammatory activity of ZBC nanoparticles was assessed to confirm their potential as an efficient capsaicin delivery system.

## 2. Results and Discussion

### 2.1. Effect of the Zein-to-BP Ratio on Nanoparticle Properties

**Figure 2:**
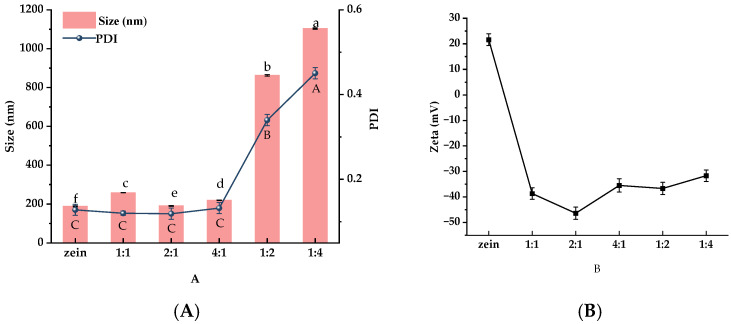
Particle size, PDI (**A**), and zeta potential (**B**) of BZC nanoparticles at different ratios a–f, A–C, Different letters indicate statistically significant differences between experimental groups (*p* < 0.05).

To optimize the wall material ratio, BZC nanoparticles were prepared at different Zein-to-BP ratios and subsequently characterized. As shown in Figure 2A, the particle size and PDI of pure Zein nanoparticles were 188.65 ± 2.54 nm and 0.134 ± 0.021, respectively. Upon the addition of BP, the particle sizes at ratios of 1:1, 2:1, and 4:1 were 258.19 ± 0.77 nm, 210.38 ± 0.83 nm, and 219.67 ± 1.34 nm, with PDI values ranging from 0.113 to 0.122. The slight increase in particle size suggests that BP was successfully attached to the Zein surface, forming a homogeneous nanoparticle system. However, when the ratios were 1:2 and 1:4, particle sizes markedly increased to 862.63 ± 3.83 nm and 1103.54 ± 3.17 nm (*p* < 0.05), with PDIs ranging from 0.339 to 0.459. These results indicate that the addition of an appropriate amount of BP had little effect on particle size, whereas excessive BP led to a significant increase, likely due to the aggregation of Zein nanoparticles.

Zeta potential is commonly used to evaluate the stability of nanoparticle systems. Generally, higher absolute values of zeta potential indicate stronger electrostatic repulsion between particles, resulting in greater colloidal stability. Conversely, lower absolute values reduce electrostatic repulsion and promote aggregation, which may eventually cause precipitation [21]. As shown in Figure 2B, pure Zein nanoparticles exhibited a positive surface charge (+21.62 mV). After the addition of BP, the nanoparticles shifted to a negative surface charge, confirming that negatively charged BP successfully coated the Zein surface and altered its charge polarity. Among the tested ratios, nanoparticles prepared at a Zein-to-BP ratio of 2:1 displayed the highest absolute zeta potential, suggesting uniform surface charge distribution and improved stability. Based on particle size, PDI, and zeta potential, the Zein-to-BP ratio of 2:1 was identified as the optimal condition for preparing stable composite nanoparticles, and this ratio was therefore selected for subsequent capsaicin (CAP) delivery studies.

### 2.2. Encapsulation Efficiency and Drug-Loading Capacity of Nanoparticles

To optimize the encapsulation performance of the nanodelivery system, Zein-to-CAP mass ratios of 2.5:1, 5:1, 10:1, 20:1, and 30:1 were compared. The particle size, PDI, zeta potential, encapsulation efficiency, and drug-loading capacity of the nanoparticles are summarized in Table 1. At Zein-to-CAP ratios of 2.5:1, 5:1, and 10:1, the particle size slightly increased, which may be attributed to the effective encapsulation of CAP and the formation of stable core-shell structures. However, when the ratio further increased to 20:1 and 30:1, the particle size decreased to 192.66 nm and 191.33 nm, respectively, suggesting that excess Zein molecules failed to interact effectively with CAP, resulting in smaller average particle sizes.

Regarding encapsulation efficiency and drug loading, nanoparticles prepared at ratios of 2.5:1, 5:1, and 10:1 achieved efficiencies of 54.03 ± 0.11%, 38.06 ± 0.87%, and 61.75 ± 1.34%, respectively, which were significantly higher than those at 20:1 and 30:1. Notably, the 2.5:1 ratio exhibited the highest drug-loading capacity, reaching 184.57 ± 0.74 μg/mg. Yin et al. [23] employed a nanoparticle-based delivery system to encapsulate CAP, achieving a drug loading of only 36.85 μg/mg, which was lower than that obtained in the present study.

Taken together, nanoparticles prepared at a Zein-to-CAP ratio of 2.5:1 displayed a smaller particle size, good homogeneity, superior encapsulation efficiency, and the highest drug-loading capacity. Therefore, this ratio was selected as the optimal formulation for the subsequent construction of BZC nanoparticles.

### 2.3. FTIR Analysis

**Figure 3:**
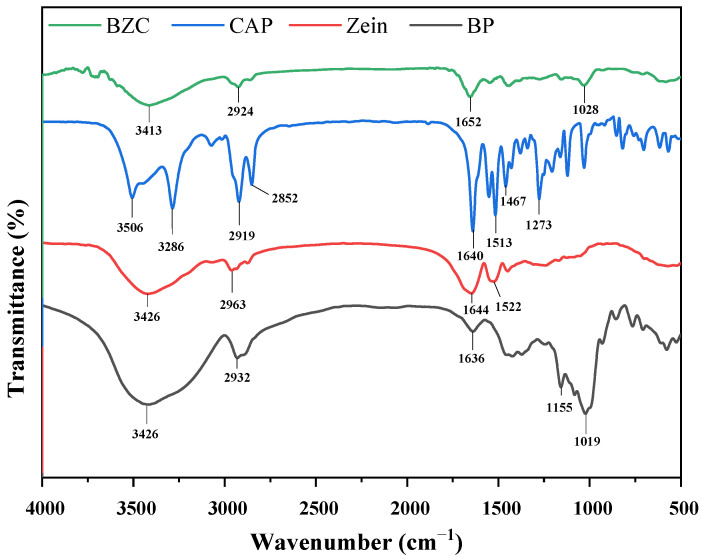
FTIR spectra of Zein, BP, CAP, and BZC nanoparticles.

FTIR spectroscopy provides insights into the characteristic functional groups of compounds, while the precise positions of the absorption peaks are also modulated by the microenvironment surrounding these groups. Thus, FTIR is a useful tool to investigate the intermolecular interactions among BP, Zein, and CAP within the nanoparticles. The spectra of individual components (BP, Zein, CAP) and composite nanoparticles (BZC) are presented in Figure 3. Zein, BP, and CAP exhibited −OH stretching vibrations at 3426, 3458, and 3286 cm^−1^, respectively, C–H stretching bands at 2963, 2932, and 2919 cm^−1^, and C=O stretching peaks at 1644, 1636, and 1640 cm^−1^. Zein and CAP showed N–H bending absorptions at 1522 and 1513 cm^−1^, respectively. Moreover, CAP displayed a characteristic aromatic band at 1467 cm^−1^ [24] and a C–O–C stretching peak at 1273 cm^−1^ [25]. In BZC nanoparticles, CAP absorption bands between 500–1640 cm^−1^ were masked by the characteristic peaks of Zein, indicating efficient encapsulation. A red shift of the -OH stretching peak to 3413 cm^−1^ suggested hydrogen bonding interactions. Additionally, slight shifts in –OH, C–H, C=O, and N–H bands in BZC indicated the presence of hydrogen bonding and hydrophobic interactions among BP, Zein, and CAP. Changes in the BP -OH band further implied that BP contributed to stabilizing the nanoparticles via hydrogen bonding and hydrophobic interactions.

### 2.4. Fluorescence Spectroscopy

**Figure 4:**
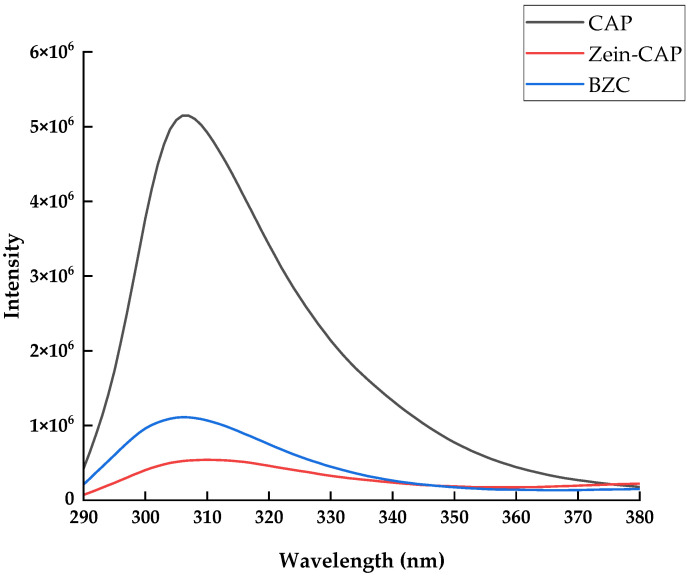
Fluorescence spectra of Zein, BP, CAP, and BZC nanoparticles.

Fluorescence spectroscopy is widely used to investigate the changes in the microenvironment of proteins induced by their interactions with other compounds. The intrinsic fluorescence of proteins mainly originates from tryptophan and tyrosine residues, whose emission intensity is closely related to the polarity of their surrounding environment. When other components interact with proteins, changes in fluorescence intensity can be observed [26]. As shown in Figure 4, the fluorescence intensity increased after the incorporation of BP, suggesting that the hydrophilic polysaccharide altered the local microenvironment of Zein, rendering it more polar [21]. Compared with free CAP, the fluorescence intensity of BZC nanoparticles decreased markedly, indicating fluorescence quenching due to the encapsulation of CAP.

### 2.5. XRD Analysis

**Figure 5:**
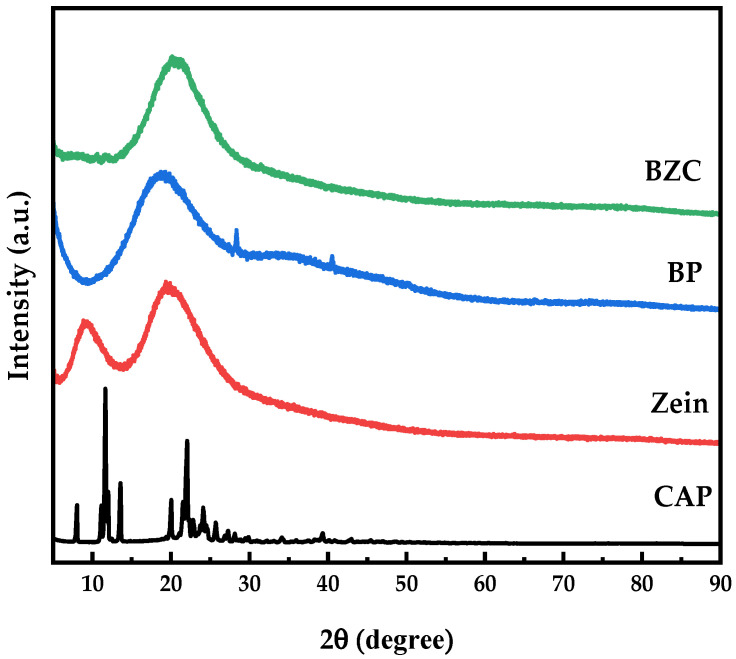
XRD patterns of Zein, BP, CAP, and BZC nanoparticles.

XRD patterns (Figure 5) revealed that Zein exhibited two broad diffraction peaks around 9.08° and 19.57°, while BP displayed a weak, broad hump near 21°, indicating the presence of microcrystalline regions [16]. In contrast, free CAP exhibited multiple sharp diffraction peaks between 5° and 30°, characteristic of a crystalline structure. Previous studies have shown that crystalline bioactive compounds often lose their crystallinity upon encapsulation [27]. In BZC nanoparticles, the crystalline peaks of CAP disappeared completely, suggesting that its ordered molecular arrangement was disrupted, leading to an amorphous state. These findings, consistent with FTIR results, confirmed the successful encapsulation of CAP in the nanoparticles.

### 2.6. Morphological Observation

**Figure 6:**
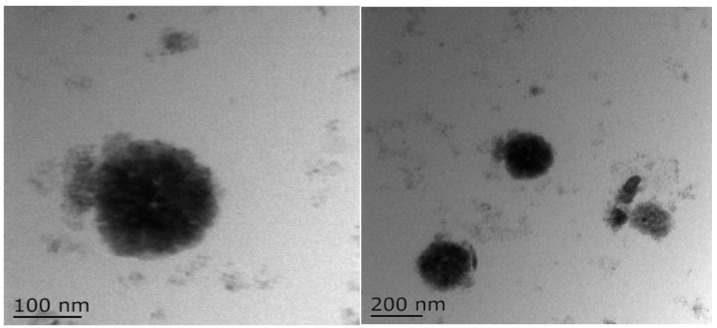
TEM images of BZC nanoparticles.

**Figure 7:**
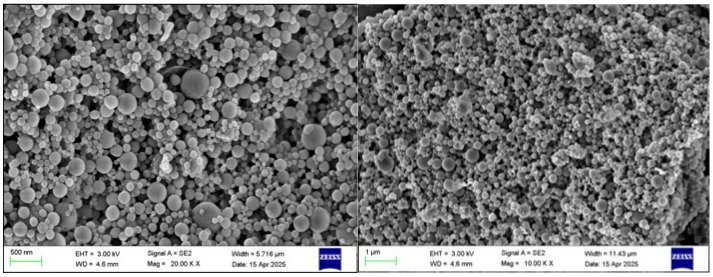
SEM images of BZC nanoparticles.

TEM images of BZC nanoparticles (Figure 6) demonstrated uniform spherical structures with well-dispersed particles, indicating that the selected Zein-to-BP ratio was appropriate and prevented aggregation. SEM images (Figure 7) further revealed smooth, spherical particles without aggregation, supporting the successful formation of stable nanoparticles.

### 2.7. Stability

#### 2.7.1. Effect of pH

**Figure 8:**
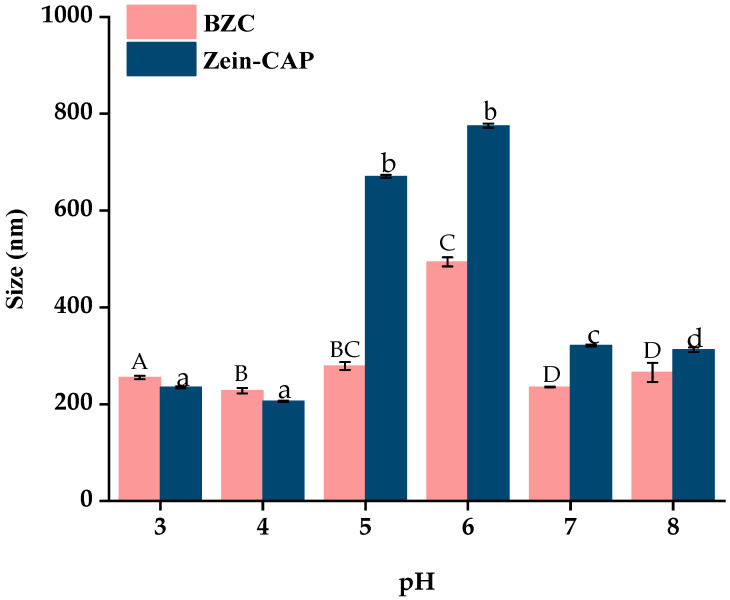
Effect of pH on the particle size of Zein–CAP and BZC nanoparticles. a–d, A–D, Different letters indicate statistically significant differences between the experimental groups’ (*p* < 0.05) storage stability.

Changes in pH may alter the physical state of nanoparticles, potentially leading to aggregation and reduced delivery performance [28]. The particle size distribution of nanoparticles at different pH values is shown in Figure 8. Zein–CAP nanoparticles were stable at pH 3–4 and 7–8, but aggregated at pH 5–6. In contrast, BZC nanoparticles maintained stable particle sizes (223.3–259.2 nm) at pH 3–5, while a moderate increase (434.6–505.1 nm) was observed at pH 5–6 without obvious aggregation. Since the isoelectric point of Zein is 6.2, nanoparticles stabilized solely by Zein are prone to aggregation near this pH due to reduced electrostatic repulsion [29]. The incorporation of BP effectively suppressed aggregation, rendering the BZC nanoparticles system more stable than Zein–CAP.

#### 2.7.2. Storage Stability Analysis

**Figure 9:**
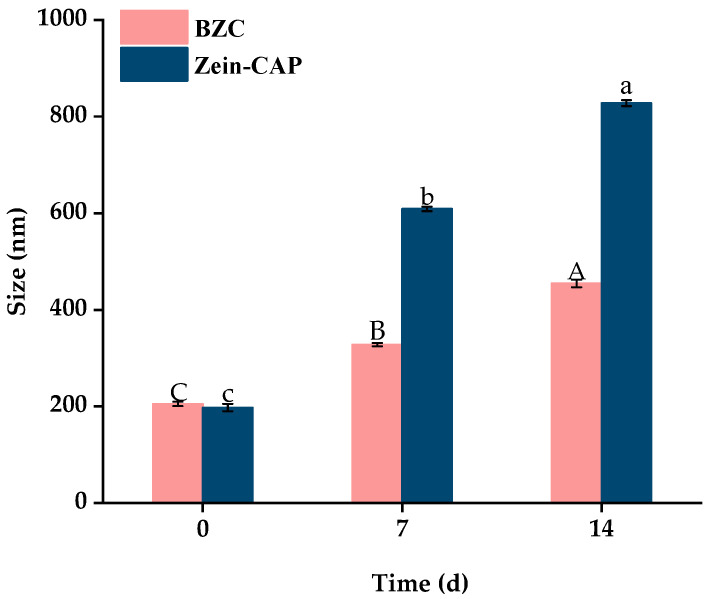

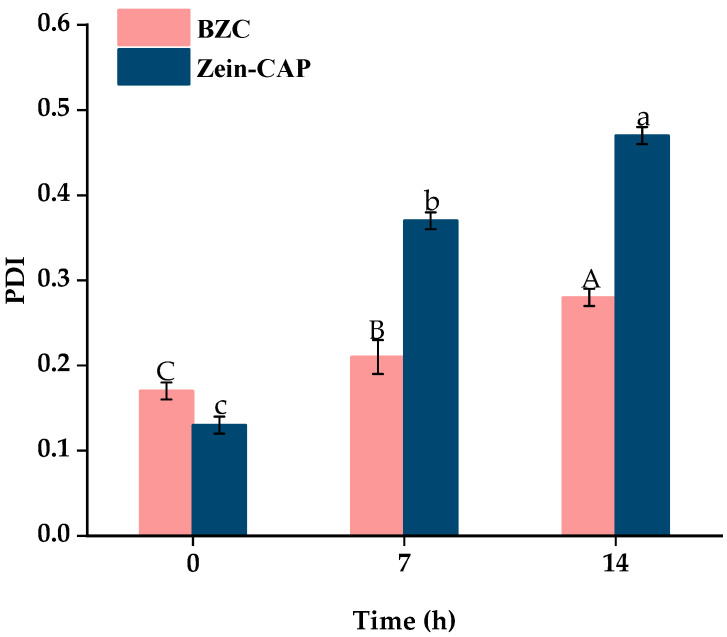
Particle size and PDI of BZC nanoparticles at 0, 7, and 14 days. A–C, a–c, Different letters indicate statistically significant differences between experimental groups (*p* < 0.05).

Storage stability is crucial for practical applications of nanoparticles. As shown in Figure 9, particle sizes of BZC nanoparticles gradually increased over 14 days at 4 °C, stabilizing at 500 nm without visible precipitation. In contrast, Zein–CAP nanoparticles showed slight aggregation after 7 days and significant sedimentation after 14 days, likely due to hydrophobic interactions among particles [27]. PDI values also increased with particle size, but BZC showed only minor changes, remaining stable at 0.28 after 14 days. No visible flocculation was observed in BZC dispersions, demonstrating superior storage stability compared to Zein–CAP.

#### 2.7.3. Ionic Strength Stability Analysis

**Figure 10:**
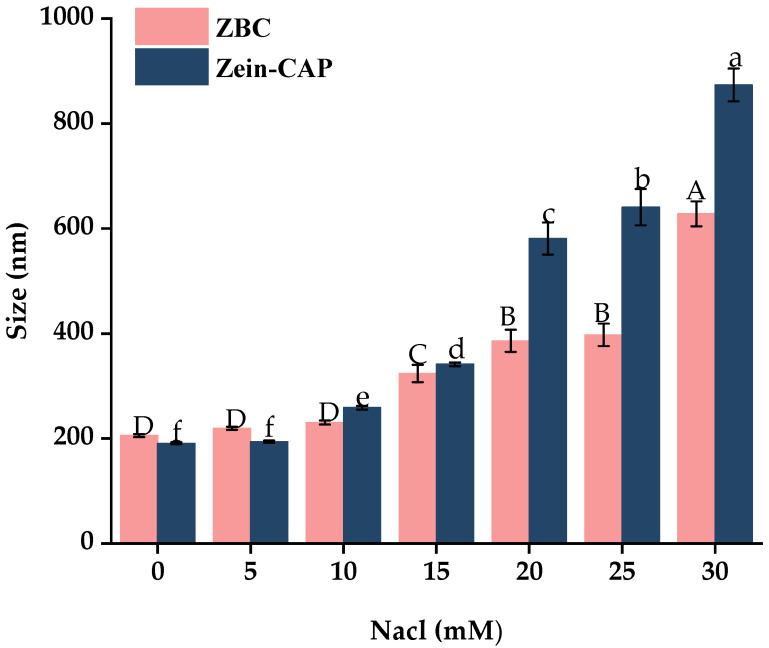
Effect of Different Ionic Concentrations on the particle size of Zein–CAP and BZC nanoparticles. a–f, A–D, Different letters indicate statistically significant differences between experimental groups (*p* < 0.05).

Ionic strength influences nanoparticle dispersion and stability [30]. As shown in Figure 10, particle size increased with NaCl concentration due to electrostatic shielding, which promotes protein nanoparticle precipitation. Zein–CAP nanoparticles began aggregating at 20 mM NaCl, while BZC remained stable until 30 mM. These results indicate that BP improved ionic strength tolerance and enhanced colloidal stability of the nanoparticles.

### 2.8. In Vitro Simulated Digestion and Release

**Figure 11:**
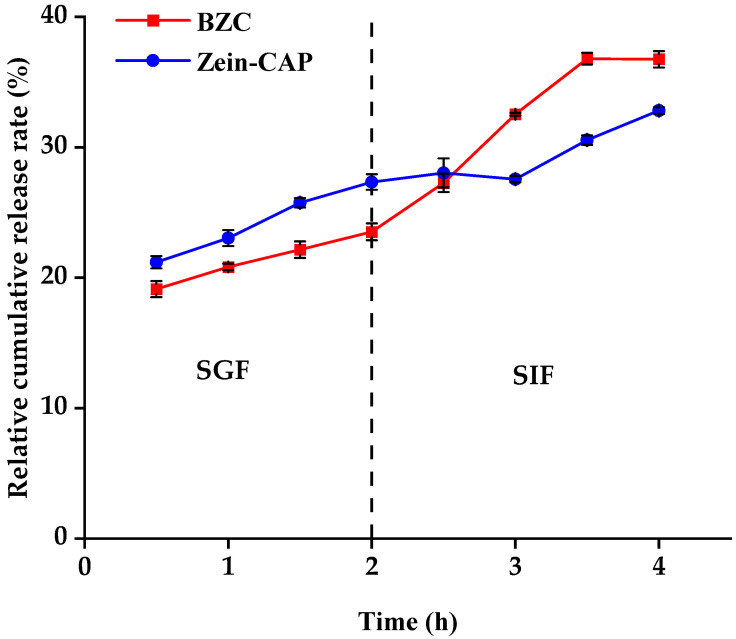
CAP digestion release characteristics in Zein–CAP and BZC nanoparticles.

The release profiles of CAP from Zein–CAP and BZC nanoparticles during simulated digestion are presented in Figure 11. In simulated gastric fluid (SGF), both systems showed slow release, with cumulative release rates of 23.52% (BZC) and 27.34% (Zein–CAP) after 2 h. Previous studies demonstrated that Zein resists pepsin digestion [31], effectively limiting CAP release. In BZC, BP coated the Zein surface, protecting its structure from enzymatic degradation and further slowing CAP release. Thus, BZC exhibited better sustained-release properties in SGF.

In simulated intestinal fluid (SIF), the release rates increased due to enzymatic degradation of Zein and BP. After 2.5 h, the release from BZC exceeded that of Zein–CAP, and after 240 min, cumulative release reached 36.76% for BZC, compared with 32.82% for Zein–CAP. These results suggest that the composite nanoparticles provided the controlled and enhanced release of CAP. Similar findings were reported by Yin et al. [23], where CAP encapsulated in thiolated chitosan-modified Zein exhibited higher cumulative release than unmodified Zein nanoparticles after 240 min of SIF digestion.

### 2.9. Effect of Nanoparticles on

Xylene is an inflammatory agent that induces ear edema in mice. Topical application immediately causes local vasodilation and increased capillary permeability, leading to acute exudative inflammation and edema in the ear. The effects of composite nanoparticles on ear edema in model mice are shown in Table 2. Compared with the blank group, the positive control dexamethasone group exhibited significantly reduced ear thickness and swelling rate with an inhibition rate of 54.7% (*p* < 0.01). In the experimental groups, low (27.17 mg/kg), medium (54.35 mg/kg), and high-dose (108.70 mg/kg) nanoparticle groups, as well as the capsaicin group (20 mg/kg), all showed significant reductions in ear edema (*p* < 0.01), with inhibition rates of 35.0%, 45.7%, 51.8%, and 41.5%, respectively. These results indicate that both the nanoparticle and capsaicin groups effectively suppressed xylene-induced ear swelling. Notably, the high-dose nanoparticle group contained the same amount of capsaicin as the free capsaicin group, but both the medium- and high-dose nanoparticle groups exhibited lower ear edema and higher inhibition rates, suggesting superior anti-inflammatory efficacy compared with free capsaicin. Moreover, the BP–Zein group also showed a slight reduction in swelling relative to the saline group, which may be attributed to the intrinsic anti-inflammatory activity of chamaemoro polysaccharides. Therefore, the anti-inflammatory effect of nanoparticles may not solely depend on capsaicin but may also result from the synergistic interaction between capsaicin and chamaemoro polysaccharides. In summary, both free capsaicin and nanoparticle formulations effectively inhibited acute inflammation, while nanoparticle delivery further enhanced the anti-inflammatory efficacy of capsaicin.

## 3. Materials and Methods

### 3.1. Chemicals

Capsaicin (CAP, 95% purity), (Jian Shengda Essential Oil Co. Ltd., Jiangxi, China). *Brassica rapa* L. polysaccharide (Mw 29 kDa), (Shaanxi Junhe Biotechnology Co. Ltd., Shaanxi, China), Zein (>92% purity), (Shanghai Yuanye Biotechnology Co. Ltd., Shanghai, China), absolute ethanol (analytical grade), (Pharmaceutical Chemistry Co. Ltd., Tianjin, China), ethyl acetate (analytical grade), (Tianjin Tianli Chemical Reagent Co. Ltd., Tianjin, China), dexamethasone (Chongqing Kerui Pharmaceutical (Group) Co. Ltd., Chongqing, China), sodium carboxymethyl cellulose (Shanghai Shanpu Chemical Co. Ltd., Shanghai, China), and xylene (analytical grade) (Sichuan Xilong Scientific Co. Ltd., Sichuan, China).

### 3.2. Preparation of Nanoparticles

#### 3.2.1. Preparation of BP–Zein Nanoparticles

Following the method described by Yin et al. [23]. with slight modifications, the preparation procedure is illustrated in Figure 2. Briefly, 300 mg of Zein was dissolved in 50 mL of 80% ethanol and stirred at 600 rpm for 30 min to obtain a Zein stock solution (DF-101S, Xingchuang Science, Shanghai, China). The resulting solution was then centrifuged at 10,000× *g* for 10 min (5810R, Eppendorf, Germany), and the supernatant was collected and reserved for subsequent experiments. Different amounts of *Brassica rapa* L. polysaccharide (BP) were dissolved in 50 mL of distilled water under constant stirring until complete dissolution. Under high-speed shearing at 4500 rpm (FJ200-SH, Huxi, Shanghai, China), the Zein solution was injected into the BP solution at a flow rate of 2 mL/min. The mixtures were prepared with Zein-to-BP mass ratios of 1:0, 1:1, 2:1, 4:1, 1:2, and 1:4. Ethanol was subsequently removed using a rotary evaporator (YT-RE-52AA, Yetuo, Shanghai, China), BP–Zein nanoparticles with different ratios were freeze-dried to obtain dry nanoparticles suitable for storage and subsequent characterization (LGJ-124, Tengfang, Shanghai, China).

#### 3.2.2. Preparation of BP–Zein–CAP Nanoparticles

**Figure 12:**
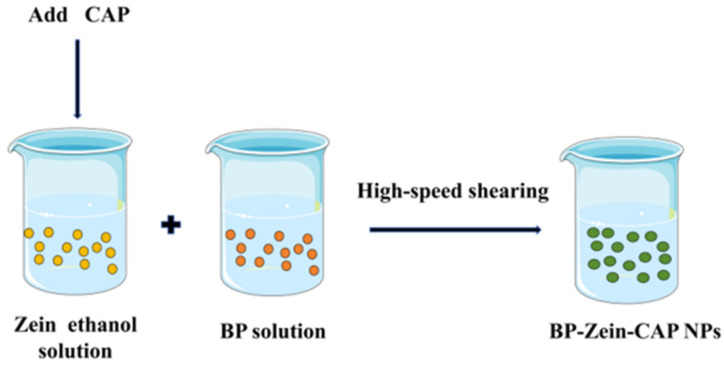
Schematic diagram of BP–Zein–CAP NP preparation.

BP–Zein–CAP (BZC) nanoparticles were prepared according to Chang et al. [32]. with slight modifications (Figure 12). To determine the optimal CAP loading, 300 mg of Zein and varying amounts of CAP were co-dissolved in 50 mL of 80% ethanol solution to obtain Zein-to-CAP mass ratios of 2.5:1, 5:1, 10:1, 20:1, and 30:1. Separately, 150 mg of BP was dissolved in 50 mL of distilled water. Under high-shear homogenization (4500 rpm), the Zein–CAP solution was injected into the BP solution at 2 mL/min. Ethanol was removed by rotary evaporation, and the suspension was centrifuged to eliminate unencapsulated CAP. The resulting BP–Zein–CAP nanoparticles were collected and freeze-dried.

For comparison, Zein–CAP nanoparticles were also prepared. Briefly, 300 mg of Zein and 120 mg of CAP were co-dissolved in 50 mL of 80% ethanol solution and injected into 50 mL of distilled water under high-shear homogenization (4500 rpm). The mixture was treated using the same rotary evaporation, centrifugation, and freeze-drying procedures as described above to obtain Zein–CAP nanoparticles.

### 3.3. Determination of Particle Size, Polydispersity Index (PDI), and Zeta Potential

The particle size, polydispersity index (PDI), and zeta potential of composite nanoparticles were determined using a laser particle size analyzer (NanoBrook 90Plus Zeta, Brookhaven Instruments, Holtsville, NY, USA). Freshly prepared nanoparticle solutions were diluted with distilled water. Prior to measurement, samples were equilibrated in the instrument for 120 s at a test temperature of 25 °C. Each sample was tested three times, and the average value was recorded.

### 3.4. Determination of Encapsulation Efficiency (EE) and Drug Loading (LC)

To determine the encapsulation efficiency, 5 mL of BP–Zein–CAP nanoparticle dispersion was mixed with 5 mL ethyl acetate under agitation to separate the free CAP. After phase separation, the upper organic phase containing free CAP was collected, diluted, and analyzed by UV–Vis (721, Jinghua Technology, Shanghai, China) at 280 nm. Encapsulation efficiency and drug loading were calculated according to the following equations:

$$\mathrm{EE}\;(\%)\;=\;\frac{M_{1}-M_{2}}{M1}\times 100\%$$

$$\mathrm{LC}\;(\mu g/\mathrm{mg})=\frac{M_{1}-M_{2}}{W}$$

where M_1_ is the total amount of CAP, M_2_ is the free CAP, and W is the weight of the freeze-dried nanoparticles.

### 3.5. Fourier Transform Infrared (FTIR) Spectroscopy

The chemical structures of BP, Zein, CAP, and BZC nanoparticles were analyzed using an FTIR spectrometer (Nicolet iS20, Thermo Fisher Scientific, Waltham, MA, USA). Samples were thoroughly mixed with dry KBr powder, ground, and pressed into transparent pellets. Spectra were recorded in the range of 400–4000 cm^−1^ with a resolution of 3.8 cm^−1^ and 32 scans per sample.

### 3.6. Fluorescence Spectroscopy (FS)

BZC nanoparticles were diluted with deionized water and measured using a fluorescence spectrophotometer (FLS1000, Edinburgh, UK) with deionized water as a blank. Fluorescence emission spectra were recorded in the range of 300–600 nm at an excitation wavelength of 280 nm with both excitation and emission slit widths set to 10 nm.

### 3.7. X-Ray Diffraction (XRD) Analysis

Powder samples of BP, Zein, CAP, and freeze-dried BZC nanoparticles were gently packed into the sample holder. The diffraction patterns were recorded using an X-ray diffractometer (XRD-6100, Shimadzu, Kyoto, Japan) with a Cu Kα radiation source (λ = 0.15406 nm, Cu filament) at 40 kV and 40 mA, with a scanning step size of 0.02°, a dwell time of 0.1 s, and a scanning range of 2θ = 5–90°.

### 3.8. Scanning Electron Microscopy (SEM)

Freeze-dried nanoparticle powders were fixed onto conductive adhesive tapes, sputter-coated with gold for 120 s, and examined using a field-emission SEM (Sigma 360, ZEISS, Oberkochen, Germany) operated at an accelerating voltage of 3 kV in secondary electron mode.

### 3.9. Transmission Electron Microscopy (TEM)

Freshly prepared nanoparticle suspensions were diluted, deposited onto carbon-coated copper grids, blotted with filter paper, and dried under an infrared lamp. Morphology was observed using a transmission electron microscope (Tecnai 12, FEI Company, Hillsboro, OR, USA).

### 3.10. Stability Tests

#### 3.10.1. pH Stability

The pH of the Zein–CAP and BZC nanoparticle suspensions was adjusted to 3.0, 4.0, 5.0, 6.0, 7.0, and 8.0 using 0.1 M HCl or NaOH. After storage overnight at 4 °C, particle size was measured using a Zeta potential analyzer.

#### 3.10.2. Storage Stability

Freshly prepared Zein–CAP and BZC nanoparticle dispersions were stored at 4 °C for 7 and 14 days. Particle size and PDI were measured at each time point.

#### 3.10.3. Ionic Strength Stability

Equal volumes (10 mL) of Zein–CAP or BZC nanoparticle suspensions were mixed with NaCl solutions of different concentrations (0–30 mM), resulting in final NaCl concentrations of 0, 10, 15, 20, 25, and 30 mM. After overnight storage at 4 °C, particle size was determined.

#### 3.10.4. In Vitro Simulated Digestion

In vitro digestion was performed according to the method of Chang et al. [32]. with minor modifications. Briefly, 10 mL of freshly prepared Zein–CAP or BZC dispersions was mixed with 10 mL simulated gastric fluid and incubated at 37 °C with shaking at 110 rpm for 120 min (THZ-D, Shuozhou, Jiangsu, China). Aliquots (2 mL) were collected every 30 min, heated at 80 °C for 5 min to inactivate pepsin, and replenished with the same volume of fresh gastric fluid. The resulting mixture (10 mL) was then combined with 10 mL simulated intestinal fluid and incubated at 37 °C with shaking at 110 rpm for 120 min. Samples (2 mL) were withdrawn every 30 min, heated at 90 °C for 5 min to inactivate trypsin, and replenished with fresh intestinal fluid. At the end of digestion, CAP was extracted with ethyl acetate, and the supernatant was diluted appropriately for CAP quantification.

### 3.11. Xylene-Induced Ear Edema in Mice

**Figure 13:**
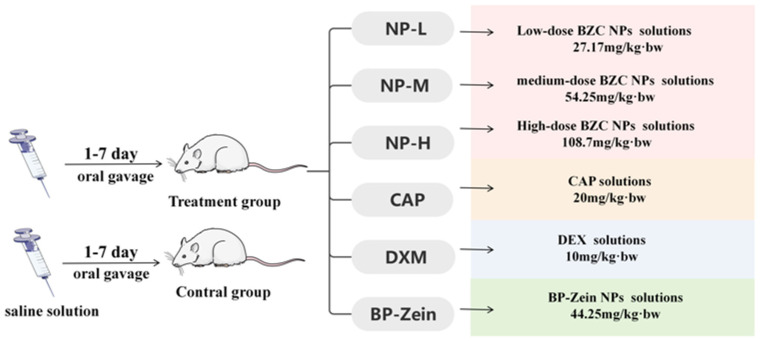
Roadmap for experiments.

The method was performed with slight modifications according to He, X. et al. [33]. Eighty-four KM mice (18–22 g, with equal numbers of males and females) were acclimated for 3 days and then randomly assigned to seven groups (*n* = 12 per group) based on body weight. The treatment groups were as follows: capsaicin group, 20 mg/kg; nanoparticle groups at low, medium, and high doses, 27.17, 54.35, and 108.70 mg/kg, respectively. BP–Zein group, 44.25 mg/kg; dexamethasone group, 10 mg/kg; and control group, receiving saline. All treatments were administered once daily by oral gavage at a volume of 0.2 mL/10 g body weight for 7 consecutive days. The experimental schematic is illustrated in Figure 13.

One hour after the final administration, inflammation was induced by applying 20 μL of xylene to both the anterior and posterior surfaces of the left ear of each mouse using a microsyringe. One hour after xylene application, the mice were euthanized by cervical dislocation. Ear punches of 8 mm diameter were collected from the same site of both ears and weighed. The swelling degree (SWD) was determined as the weight difference between the left and right ear punches, and the inhibition rate (%) was calculated using the following equations.This animal study was conducted in accordance with the rules of the Animal Welfare and Ethics Committee of Xinjiang Agricultural University (Urumqi, Xinjiang, China). Detailed information can be found in the Supplementary file.

$$\mathrm{Swelling}\;\mathrm{Degree}(\mathrm{mg})=W_{1}-W_{2}$$

$$\mathrm{Inhibition}\;\mathrm{rate}(\%)=\frac{\mathrm{Mean}\;\mathrm{swelling}\;\mathrm{of}\;\mathrm{control}\;\mathrm{group}-\mathrm{Mean}\;\mathrm{swelling}\;\mathrm{of}\;\mathrm{treated}\;\mathrm{group}}{Mean\;swelling\;of\;control\;group}$$

where W_1_ represents the weight of the left ear punch and W_2_ represents the weight of the right ear punch.

### 3.12. Statistical Analysis

All experiments were conducted in triplicate, and data were expressed as the mean ± standard deviation (SD). Statistical analyses were performed using SPSS software (version 27.0, IBM, Chicago, IL, USA). Intergroup differences were assessed using one-way analysis of variance (ANOVA) for multiple comparisons, followed by the Student’s *t*-test for pairwise comparisons. A *p* < 0.05 was considered statistically significant, while a *p* < 0.01 indicated highly significant differences. Graphs were plotted using Origin (version 2024, OriginLab, MA, USA).

## 4. Conclusions

In conclusion, an efficient capsaicin delivery system, BP–Zein–Capsaicin (BZC) nanoparticles, was successfully developed. The nanoparticles exhibited high drug loading, excellent stability, and sustained-release properties. In vivo anti-inflammatory experiments revealed that the nano-delivery system significantly enhanced the anti-inflammatory activity of capsaicin. The optimized structural characteristics of the nanoparticles contributed to the improvement of their biological activity. This study provides new insights and strategies for enhancing the stability, bioavailability, and potential application of capsaicin in functional foods.

## Figures and Tables

**Table 1:** Characterization of BZC nanoparticles (mean ± SD).

Zein:CAP	Particle Size (nm)	PDI	ζ-Potential (mV)	EE (%)	LC (μg/mg)
2.5:1	203.05 ± 2.71 ^b^	0.138 ± 0.02 ^a^	−44.9 ± 1.8 ^a^	54.03 ± 0.11 ^b^	184.57 ± 0.74 ^a^
5:1	209.52 ± 1.69 ^b^	0.129 ± 0.004 ^b^	−44.4 ± 0.9 ^a^	38.06 ± 0.87 ^c^	68.48 ± 1.56 ^b^
10:1	243.36 ± 1.25 ^a^	0.109 ± 0.01 ^b^	−40.5 ± 1.2 ^b^	61.75 ± 1.34 ^a^	57.89 ± 1.22 ^c^
20:1	192.66 ± 1.47 ^c^	0.128 ± 0.01 ^b^	−43.6 ± 2.2 ^a^	33.21 ± 0.86 ^cd^	16.20 ± 0.42 ^d^
30:1	191.33 ± 2.04 ^c^	0.116 ± 0.003 ^b^	−42.0 ± 1.5 ^ab^	31.85 ± 2.59 ^d^	10.68 ± 0.86 ^e^

a–e, Different letters indicate statistically significant differences between experimental groups (*p* < 0.05).

**Table 2:** Effect of BZC nanoparticles on xylene-induced ear swelling in mice.

Group	Swelling Degree (SWD, mg)	Inhibition Rate (IR, %)
Control	7.92 ± 1.03	-
CAP	4.63 ± 1.61 **	41.5%
DXM	3.59 ± 1.66 **	54.7%
NP-L	5.15 ± 1.28 **	35.0%
NP-M	4.30 ± 1.28 **	45.7%
NP-H	3.82 ± 1.35 **	51.8%
BP–Zein	7.14 ± 1.13	15.8%

Note: Values are expressed as mean ± SD (*n* = 12). ** *p* < 0.01 compared to the control group.

## Data Availability

The original contributions presented in the study are included in the article/Supplementary Materials, further inquiries can be directed to the corresponding author.

## Supplementary Materials

The following supporting information can be downloaded at: https://www.mdpi.com/article/10.3390/molecules30224459/s1.

## Abbreviations

The following abbreviations are used in this manuscript:

BP	Brassica rapa L. polysaccharide
CAP	Capsaicin
Zein–CAP	Capsaicin-loaded Zein
BZC	Capsaicin-loaded Brassica rapa L. polysaccharide–Zein
PDI	Polydispersity index
FTIR	Fourier transform infrared spectroscopy
XRD	X-ray diffraction
SEM	Scanning electron microscope
TEM	Transmission electron microscopy
SGF	Simulated gastric fluid
SIF	Simulated intestinal fluid
UV–Vis	UV-visible spectrum
LC	Load volume
EE	Encapsulation rate
SWD	Swelling degree
IR	Inhibition rate
