# Supplementary material for: Preparation and Characterization of Brassica rapa L. Polysaccharide–Zein Nanoparticle Delivery System Loaded with Capsaicin
Source: Molecules. 2025 Nov 19;30(22):4459. doi: 10.3390/molecules30224459 (PMC12655759; doi:10.3390/molecules30224459)
Supplement: Supplementary file 1 [file molecules-30-04459-s001.zip › molecules-3949798-supplementary.pdf]

实验动物福利伦理审查申请书

Application of Welfare and Ethics Approval for Research involving Animals

申请日期：2023 年 9 月 23 日

课题名称，编号，来源：自治区重点研发项目课题-农科院(实验室-果品采后)(2022B02037-2)

项目负责人	职称	所在院所
杨晓君	教授	新疆农业大学食品科学与药学学院

联系电话：15276826522	E-mail：xjxiaojuny@163.com
申请目的	初次申请（√） 延长（ ） 修改原申请（原批准号：________）（ ）

动物实验名称（中文和英文）：

负载辣椒素的恰玛古多糖-玉米醇蛋白纳米颗粒的急性毒性实验及其抗炎作用

Acute toxicity test of capsaicin-loaded *Brassica rapa* L.polysaccharide-zein nanoparticles and their anti-inflammatory effect

膳食纤维与α-淀粉酶抑制剂微胶囊驼奶片的急性毒性实验及其体内调节血糖作用

Acute Toxicity Study of Dietary Fiber and α-Amylase Inhibitor Microencapsulated Camel Milk Tablets and Their In Vivo Blood Glucose-Regulating Effects

拟实验日期	2025 年 5 月 1 日至 2025 年 9 月 1 日

序号	实验执行人	职　称	所在院所	联系电话
1	杨晓君	教　授	新疆农业大学食品科学与药学学院	15276826522
2	袁蜜	研究生	新疆农业大学食品科学与药学学院	18152932537
3	陈乐乐	研究生	新疆农业大学食品科学与药学学院	19945812661

实验动物来源	新疆医科大学实验室动物中心
实验动物许可证和质量合格证明编号	SCXK（新）2022-0002；

品种、品系	年龄或体重	等级	性别	数量	其它
昆明小鼠	20g	无菌级	雌	72	
昆明小鼠	20g	无菌级	雄	72	
ICR 小鼠	20g	无菌级	雄	120	
BALB/c 小鼠	20g	无菌级	雄	84	

一、概述本实验的目的及对人类、动物或科学的贡献

实验目的为研究负载辣椒素的恰玛古多糖-玉米醇蛋白纳米颗粒的急性毒性实验及其体内抗炎作用；膳食纤维与α-淀粉酶抑制剂微胶囊驼奶片的急性毒性实验及其体内调节血糖作用

二、请以实验动物“3R”原则（实验动物替代、减少和优化）为考虑重点，说明进行动物实验

的必要性，包括非动物模型不合适性及选择该动物品种的理由。

1、使用动物的理由（在括号内打“√”）：

（√）（1）一些生物学过程和机理不能在体外研究

（√）（2）已进行体外实验，现须进行体内实验

（ ）（3）体外实验需要动物组织

（ ）（4）其它

请具体说明：体外细胞及酶学模型虽可提供部分机制信息，但无法系统反映体内吸收、代谢、炎症反应及血糖调控，因而不能替代整体动物实验。分别选用 BALB/c 小鼠用于抗炎实验、ICR 小鼠用于血糖调节实验、KM 小鼠用于急性毒性实验及二甲苯致鼠耳肿胀实验，均为经典实验动物模型，具有针对性和代表性，能在保证科学性与可靠性的同时提高实验结果的可信度。

2、请说明使用动物数量的充分理由：

①负载辣椒素的恰玛古多糖-玉米醇蛋白纳米颗粒的急性毒性实验及其抗炎作用

急性毒性实验：选取 30 只小鼠（雌雄各半）。分为对照组及样品组，每组 10 只。二甲苯致鼠耳肿胀实验：选取 84 只 KM 小鼠（雌雄各半）随机分为 7 组：空白组、阳性对照组、低剂量组、中剂量组、高剂量组、原药组（CAP）、BP-zein 组，每组 12 只。抗炎实验：选取 84 只雄性 BALB/C 小鼠随机分为 7 组：空白组、模型组、阳性对照组、低剂量组、中剂量组、高剂量组，BP-zein 组，每组 12 只。

②膳食纤维与α-淀粉酶抑制剂微胶囊驼奶片的急性毒性实验及其体内调节血糖作用

急性毒性实验：选取 30 只小鼠（雌雄各半），分为对照组及样品组，每组 10 只。STZ 诱导二型糖尿病调节血糖实验：将拟建模 120 只小鼠按体重随机分成 2 组，12 只小鼠给予维持基础生长活动饲料量，作为正常组，其余作为模型组。将模型组造模成功小鼠随机分为 8 组每组 12 只，分别是模型组、阳性对照组（二甲双胍组）、膳食纤维组、微胶囊组、驼乳粉组、奶片低剂量组、奶片中剂量组、奶片高剂量组。加正常小鼠组共 9 组。

设置多个实验动物分组目的在于验证功能性食品不同剂量功效，及实验造模情况。动物数量依据统计学功效、个体差异及实验重复性需要确定，在保证结果科学可靠的前提下已尽量减少使用。

三、描述动物实验的设计

1、简述实验过程（包括实验基本过程；说明动物保定的必要性，动物保定的方法，包括设备和药物；实验过程中动物有无疼痛，如果有强烈疼痛如何减轻：麻醉药名称、剂量、给药途径和维持时间；实验采集的标本用什么方法检测什么指标。）

①负载辣椒素的恰玛古多糖-玉米醇蛋白纳米颗粒的急性毒性实验及其抗炎作用

急性毒性实验：给药后短期内观察并记录体重、死亡率。抗炎实验：建立二甲苯致鼠耳肿胀/脂多糖诱导全身炎症小鼠模型，同时灌胃给予不同受试物，每只鼠每天灌胃体积不超过

0.25ml，为期8天。

②膳食纤维与α-淀粉酶抑制剂微胶囊驼奶片的急性毒性实验及其体内调节血糖作用

急性毒性实验：给药后短期内观察并记录体重、死亡率。调节血糖试验：建立二型糖尿病小鼠模型，同时灌胃给予不同受试物，每只鼠每天灌胃体积不超过0.25ml，为期28天。

动物保定的必要性：在实验过程中应尊重动物习性并善待动物，结合操作需要选择简便可靠的保定方法（捉拿保定小鼠），保证给药、采血等操作准确顺利，减少动物应激与损伤，同时注意实验人员的安全防护。

实验过程疼痛较少，减轻疼痛的方式采用以戊巴比妥（50 mg/kg）麻醉小鼠使其镇静，

实验主要采集小鼠眼眶血、小鼠肝脏、肾脏、肺、胰腺；检测小鼠体重、脏器指数、血清及脏器中的各项指标。

2、实验之后动物如何处理？（在括号内打"√"）

（ ）实验后动物可以正常存活，回归正常饲喂

（√）实验结束时动物已经无痛死亡，尸体无公害化处理

（ ）动物不能正常存活，进行安乐死（安乐死方法、使用药物及其剂量）：

声明：

我承诺该申请使用表的内容准确无误。

我同意遵守中华人民共和国国家科学技术委员会制定的《实验动物管理条例》、中华人民共和国科学技术部发布的《关于善待实验动物的指导性意见》。

我承诺包括我自己在内的该申请使用表中提及的与实验动物有接触的人员，已经参加了新疆农业大学实验动物中心要求的相关培训，掌握了申请使用表中涉及的动物实验方法，都有能力完成动物实验，并且深知使用这些活体动物及动物组织所存在的风险。

项目负责人签字：[signature]　　2023年 9月 23日

申报单位意见：

同意

主管领导签字（单位章）：[signature]　2023年 9月 25日

新疆农业大学……与药学院　6501030127624

审查主要依据：

1、该项目是否必须用实验动物进行实验，即能否用计算机模拟和细胞培养等非生命方法替代实验动物或用低等实验动物替代高等实验动物进行实验。

2、表中所填实验相关人员资格和实验相关单位是否合适。

3、表中所填实验所用实验动物的品种品系、质量等级、规格是否合适，能否通过改良设计方案或用高质量的实验动物来减少所用实验动物的数量。

4、能否通过改进实验方法、调整实验观测指标、改良处死实验动物的方法，来优化实验方案、善待实验动物。

审查	实验执行人员资格：　符合要求（√）　不符合要求（ ）

动物实验方案：适当（√） 不适当（ ）

审批意见：

（√）同意

（ ）稍作修改，同意

（ ）修改后，再次会议讨论

（ ）不同意

新疆农业大学实验动物福利伦理委员会

Animal Welfare and Ethics Committee of Xinjiang Agricultural University

2023年 9月 30日

批准号<br>Animal protocol number	2023056

说明：

1、请在实验开始前 1-2 个月提交电子版申请材料到新疆农业大学实验动物福利伦理委员会，接受审查；

2、得到审查反馈意见并修改后，递交电子版和纸质版申请书 2 份（项目负责人签字，申请院所主管科研领导签字、盖章，双面打印）。

3、动物实验程序审核批准后将给予批准号。
